# Molecular and Genetic Factors Involved in Olfactory and Gustatory Deficits and Associations with Microbiota in Parkinson’s Disease

**DOI:** 10.3390/ijms22084286

**Published:** 2021-04-20

**Authors:** Melania Melis, Antje Haehner, Mariano Mastinu, Thomas Hummel, Iole Tomassini Barbarossa

**Affiliations:** 1 Department of Biomedical Sciences, University of Cagliari, Monserrato, 09042 Cagliari, Italy; melaniamelis@unica.it (M.M.); mariano.mastinu@unica.it (M.M.); 2 Smell and Taste Clinic, Department of Otorhinolaryngology, Technical University of Dresden, 01307 Dresden, Germany; Antje.Haehner@uniklinikum-dresden.de (A.H.); thomas.hummel@tu-dresden.de (T.H.)

**Keywords:** Parkinson’s disease, smell, taste

## Abstract

Deficits in olfaction and taste are among the most frequent non-motor manifestations in Parkinson’s disease (PD) that start very early and frequently precede the PD motor symptoms. The limited data available suggest that the basis of the olfactory and gustatory dysfunction related to PD are likely multifactorial and may include the same determinants responsible for other non-motor symptoms of PD. This review describes the most relevant molecular and genetic factors involved in the PD-related smell and taste impairments, and their associations with the microbiota, which also may represent risk factors associated with the disease.

## 1. Introduction

Parkinson’s disease (PD) is a chronic neurodegenerative disorder with a prevalence of 1% in subjects aged 60–69, increasing to 3% in those over 80 years of age [1]. Pathologically, the disease is characterized by dopaminergic neuronal loss in the substantia nigra, and it is associated with intracellular inclusions, called Lewy bodies, in the neurons of affected brain regions. The Lewy bodies are intra-cytoplasmic eosinophilic deposits of a misfolded protein, α-synuclein which spreads to different regions of the brain in a prion-like fashion, giving rise to the successive non-motor and motor symptoms [2,3,4]. Clinically, PD is characterized by the presence of motor symptoms such as bradykinesia (slow movement), rigidity, tremor, postural instability (balance problems), difficulty with walking, and coordination. In addition, PD is also characterized by the occurrence of several non-motor symptoms such as sleep disturbances, apathy, anxiety, autonomic dysfunction, gastrointestinal dysfunction (such as nausea, dysphagia, abnormal salivation, constipation and defecatory dysfunction [5,6]), cognitive impairment, olfactory and gustatory dysfunctions [7,8]. Specifically, olfactory dysfunction is accepted to be an early biomarker of the disease since precedes the occurrence of clinical motor symptoms [9], with an incidence raging between 50% and 96% [10,11,12]. Although the occurrence of taste dysfunctions in PD is less clear, they have been described as non-motor manifestations of the early stages of PD [13,14,15], highlighting their role in the diagnosis of the disease.

Taste and olfaction play a key role in individuals’ behaviors, their interactions with the environment and memory processes [16], furthermore, they represent the most important factors influencing food preferences and therefore eating behavior and diet [17,18]. In fact, they cooperate and enables organisms to distinguish nutrient-rich food from noxious substances, and acts as a final checkpoint for food acceptance or rejection behavior [19,20]; therefore, it is not surprising that disorders in these two sensory modalities can have significant effects on the life’s quality [21].

The purpose of this review is to summarize the knowledge about smell and taste disorders in idiopathic PD, describing the most relevant molecular and genetic factors involved in the smell and taste impairments related with PD. We also describe the principal associations with microbiota. Accounting for these factors, could lead to a more precise assessment which would greatly help clinicians in the early diagnosis of PD.

## 2. Functions and Dysfunctions of Taste

### 2.1. Taste System

In the common language, the word “taste” is often used to describe sensations arising from the oral cavity. However, in biology the sense of taste includes all sensations mediated by a chemosensory gustatory system specialized anatomically and physiologically [22]. The molecular mechanisms underlying the perception of taste include the reception and signal transduction mechanisms, which play important roles in the oral cavity and also in a diversity of tissues including the respiratory and gastrointestinal tracts, kidney and even brain [23].

Taste sensation begins with the activation of taste receptor cells (TRCs), which are organized in taste buds located mostly on the superior surface of the tongue. The reception and transduction mechanisms of taste stimuli are located at the chemosensory apical tip of the TRCs. The generated signals are transmitted, via three cranial nerves (CN) (facial, VII; glossopharyngeal, IX and vagus, X), to the rostral part of the solitary tract nucleus (NST) of the medulla. The projections from the NST include parabrachial nucleus, thalamus (ventral posteromedial nucleus), gustatory areas of the cortex in the insula, amygdala, hypothalamus and basal ganglia [24].

It is generally assumed that human taste sensations can be divided into five qualities: bitter, sour, salty, sweet, and umami. Recently, the fat taste has been acknowledged as a sixth primary sensory quality [25,26,27,28].

### 2.2. Chemoreceptors, Receptor Genes and Taste

Chemoreceptors of the plasma membrane of TRCs interact with specific chemical stimuli to initiate an afferent signal to the brain, which results in taste perception. Specialized chemoreceptors mediate specific coding mechanisms for different taste stimuli and provide the basis for discrimination across taste qualities [29]. In humans, the chemoreception of sweet, umami, and bitter taste involves membrane proteins from the TAS1R and TAS2R families, which belong to a superfamily of G protein–coupled receptors (GPCRs). Various receptors for detection of long chain fatty acids have been proposed [30,31], including CD36 [27,32,33]. Candidate chemoreceptors have been suggested for salty and sour taste qualities [34,35,36,37,38]. The existence of several chemoreceptors reflect the importance of distinguishing beneficial from harmful chemicals of the environment [39].

Progress with understanding of the interaction between taste stimuli and chemoreceptors and the identifying of patterns of their expression in taste cells sheds light on coding of taste information by the nervous system [22]. Variations in taste receptor genes affect expression and function of taste receptors, and therefore influence taste function [40]. It is well known that variation in taste receptor genes can result in differences of the sweet, umami and bitter perception, while less is known about the genetics of sour and salty taste [41]. The *TAS2R38* gene, codifying for receptor binding the bitter-tasting thiourea compounds such as PROP and PTC, is one of the most studied in this field [42]. Variations in *TAS2R38* gene greatly contribute to the thiourea taster groups: super-tasters, medium tasters and non-tasters [43,44].

### 2.3. Extra-Gustatory Taste Receptors

As stated above, taste receptors and signal transduction molecules for sweet, bitter, and umami tastes are expressed not only in TRCs of oral cavity, but also in cells of a variety of extra-oral tissues throughout the body, including the brain [23]. The functions of this internal chemoreceptors have only been partially elucidated. However, it possible to state that they detect chemical compounds of internal environment and that modifications of this internal chemo-sensation can affect physiological functions [45]. Specifically, TAS2Rs, which detect bitter compounds, mediate several non-tasting functions and their genetic variants are associated with diverse disorders [46]. Brain neurons have been shown to respond to different chemicals [47]. Bitter TAS2Rs are expressed in multiple regions of the rat brain, TAS2R4, TAS2R107 and TAS2R38 are expressed in the brain stem, cerebellum, cortex, and nucleus accumbens and calcium signaling showed the functionality of T2R4 expressed in these cells [48].

### 2.4. Taste Dysfunction in Neurogenerative Disease (Overview)

Taste dysfunctions are described as ageusia (complete loss of taste), hypogeusia (partial loss of taste), parageusia (inadequate or wrong taste perception) and phantogeusia (presence of a persistent and unpleasant taste) [49]. Taste disorders are generally associated to medical conditions, pharmacologic or surgical interventions, exposure to toxic chemicals, head injury, advanced age or neurodegenerative diseases [14,50,51,52,53,54,55,56,57,58]. Over recent years, the link between taste dysfunctions and neurodegenerative disorders has increasingly been recognized. Some authors showed patients with Alzheimer’s disease (AD) to reporte significant reduction of taste function, by showing an increase of the detection threshold of the four basic tastes (sweet, salty, sour, and bitter) [53,54]. However, others reported no difference in detection threshold of sucrose [59,60] and sour [59] or total absence of taste alterations [61]. In a case study, Petzold et al. [62] indicated that patients with amyotrophic lateral sclerosis (ALS) reported a persistent bitter or metallic taste (phantogeusia), although no hypogeusia for taste qualities were observed. Tarlarini and colleagues showed reduction of taste and its negative consequences on psychological status and quality of life in ALS patients [63]. Taste disorders also have been described as a prominent early feature in Creutzfeldt–Jakob disease (CJD) which is one of prion diseases, a group of neurodegenerative disorders, characterized by accumulation of abnormal prion proteins in the central nervous system. In 2001, Reuber and colleagues describe for the first time a patient with CJD whose first symptoms included deficits of taste and smell [64].

### 2.5. Taste Impairments in PD

In recent years several studies evaluated gustatory function in PD patients [14,50,51,65,66,67,68], but reporting inconsistent results. This may because they were carried out by using small sample size or different assessment methods: whole mouth test (WMT), supra-threshold taste solutions sprayed into the oral cavity [69]; taste strip test, (TST), in which patients had to identify a taste from a taste strip [70,71] and electrogustometry (EGM), rapid measure of taste threshold by using electric current as stimulus) [72,73].

Despite the different tests adopted by the research groups, it is generally reported that taste can be affected in PD patients by showing persistent, but slight and stable taste impairments [74]. In particular, most of the studies identified a reduced taste sensitivity with an estimated frequency between 9% and 27% [14,50,51,52]. Shah et al. [51], using EGM, found that about 27% of PD patients had an impaired taste function. Taste thresholds measured in the front and back of the tongue were higher in PD patients, than in healthy controls (HC), suggesting significant deficits in CN VII and CN IX. Deeb et al. [50] by using EGM showed that about 22% of PD patients had impaired taste function. Kim et al. [14] by using TSTs reported a decrease in the ability to identify tastants in female but not in male PD patients when compared to HC. Cecchini et al. [68] reported difference between PD patients and HC in taste performance assessed by the TST, but not by WMT. In fact, only the TST score was significantly lower in PD patients than HC. The reason of the fact that WMT do not show reduction of taste could be due to the use of stimuli at supra-threshold concentration, which are not able to capture slight impairment of taste function.

Doty et al. [13] studied whole-mouth (WMT) and regional taste perception of early-stage PD patients and HC matched on the basis of age, sex, and race. They reported that the WMT scores were lower in the PD patients than in controls (for all four taste stimuli), and the intensity ratings for the weaker concentrations of all stimuli, except caffeine, tended to be higher in the PD patients than in HC. This last finding is consistent with the findings of Sienkiewicz- Jarosz and co-workers who demonstrated that, in the WMT test, PD patients rated quinine [65] and sucrose as more intense than HC [66]. Moreover, Doty et al. [13] using regional tests showed that subjects tended to better identify and rate the stimuli as more intense on the front than in the back of the tongue with respect to controls. These findings suggest that the suprathreshold measures of taste function are influenced by PD which differentially influences taste function on CN VII and CN IX. These results are not observed if the taste techniques are limited to WM. In addition, in the same study [13] EGM was not able to observe differences between the PD patients and controls. In addition, a reduced identification of sweet [75], salty or bitter stimuli was found [13]. Despite the slightly controversial results, it appears that taste is affected in PD, although less frequently than smell. However, future investigations are necessary to explore the causes of taste impairments related to PD.

It is interesting to note that the taste loss has been related mostly to the advanced stages of the disease [50], whereas reports on prodromal presentation are rare. Pont-Sunyer and colleagues [76] observed that the time of the taste loss onset varied between 2 and 10 years before diagnosis. Taste loss was present before the onset of motor symptoms in more than 70% of PD patients, providing evidence for a very-early onset of taste loss, which is comparable to that of olfactory impairments. Therefore, the evaluation of the taste function may be used in combination with that olfactory as a potential marker of PD. However, it is known that anosmics are more poorly able to taste than normal persons [77,78,79].

### 2.6. Role of Taste Receptors in PD

The role of taste and smell receptors in PD has been investigated showing that the cortical olfactory receptors (ORs) and the TAS2Rs are altered in PD patients [80]. Olfactory receptors OR2L13, OR1E1, OR2J3, OR52L1, and OR11H1 and taste receptors TAS2R5 and TAS2R50 were downregulated, whereas TAS2R10 and TAS2R13 were upregulated, at premotor and parkinsonian stages, in the frontal cortex area 8 of the brains in PD patients [80]. These findings support the idea that ORs and TA2SRs in the cerebral cortex may have physiologic functions that are affected in PD patients. The identification of altered regulation of OR and TAS2R in PD patients, suggests the study of the chemical signaling system of the brain to understand the mechanisms involved in the occurrence of the neurodegenerative diseases. Future studies will have to point out whether the altered TAS2R may play a role in the inflammatory mechanisms associated with the initiation of misfolding of the α-synuclein cascade.

### 2.7. Relationships between TAS2R38 and Taste Dysfunction in PD

TAS2R38 has been associated with a variety of non-tasting physiological mechanisms [17,42,46,81,82,83,84,85]. The allelic diversity of the gene codifying for TAS2R38 results in three non-synonymous coding single nucleotide polymorphisms (SNPs), which give rise to two major variants: the functional form containing proline, alanine and valine (haplotype named PAV) and the non-functional variant containing alanine, valine and isoleucine (haplotype named AVI) [44,86]. TAS2R38 SNPs dictate individual differences in PTC/PROP tasting [44,87,88], food linking patterns [82,89] and also in TAS2R38‒mediated pathophysiology [46], such as susceptibility, severity, and prognosis of upper respiratory infection, rhinosinusitis and biofilm formation in chronic rhinosinusitis patients [90,91,92,93,94,95,96,97], development of colonic neoplasm [98,99,100], taste disorders [101], and neurodegenerative diseases [102].

In the following paragraphs, we focus on TAS2R38 polymorphisms, the relative ability to perceive the bitter taste of thiourea compounds and its association with microbiota, as a genetic risk factors for development of PD.

Moberg and colleagues were the first that examine PTC sensitivity in PD patients and HC to determine whether taster status can be a marker for PD. They showed significant differences in the distribution of taster and non-taster subjects between the PD patients HC. They showed that only 44% of PD patients could detect the bitterness of PTC, as compared to 75% of HC [103]. Cossu et al. [102] confirmed the result showing a reduced of PROP taste sensitivity in PD patients compared to HC. Specifically, a decreased perceived taste intensity and reduced ability to recognize bitter-taste quality was found. They also showed an increase in the frequency of the PD patients classified as PROP non-tasters (54.13%) and a decrease in frequency of PD patients classified as PROP super-tasters (8.25%) compared to HC. Furthermore, the results showed that the homozygous genotype for the tasting variant of TAS2R38 (PAV) was uncommon in PD patients, only 5% of them carried this genotype, whereas most of them carried the non-taster form (AVI). These results seem to indicate that individuals who have a couple of tasting haplotypes (PAV/PAV) at TAS2R38 may be at lower risk of developing PD, with respect to those with the haplotype (AVI). Therefore, the latter might represent a prodromal genetic marker for the identification of early pre-degenerative changes that could be instrumental to understand the origin of this disorder. Thus, studying the PROP phenotype and genotype may represent a new, simple way to identify increased predisposition for PD.

### 2.8. Role of Microbiota on Relationships between TAS2R38 and Taste Dysfunction in PD

PD has been associated with the dysbiosis of gut microbiota [104] and imbalance in gut microbiota plays an important role in worsening of disease [105,106,107,108]. Specific taste receptors, expressed in the lower gastrointestinal tract (GI), respond to change of the composition of gut microbiota and regulate immune responses against pathogens [46,109,110,111]. In particular, it is known that when TAS2R38 expressed in the enteroendocrine cells of the gut is activated by bacterial molecules, it increases the release of β-defensin (an anti-microbial compound) [46] and a peptide hormone termed cholecystokinin (CCK). This hormone can limit the absorption of dietary toxins [112,113], inhibit feeding behavior and gastric function [114,115,116] and it also can play a key role in regulating the immune response to antigens and bacterial toxins [117]. Thus, the response of TAS2R38 represents an important defense of the organism in contrasting the noxious effects in the gut lumen.

Vascellari et al. [118] showed that the composition of the gut microbiota was different across genotypes of TAS2R38 in PD patients. Specifically, a decrease in bacteria alpha-diversity with a predominant reduction of *Clostridium* genus was associated with AVI/AVI genotype, compared to the PAV/PAV genotype. It is important to mention that some members of *Clostridium* genus produce toxin [119], while other members confer beneficial effects which has a multitude of metabolic function in the GI tract, such as modulation of gastrointestinal motility, barrier integrity and immune response [119,120,121]. Therefore, a decrease in the abundance of helpful-*Clostridium* molecules associated to a high frequency of the form of TAS2R38 receptor at a low affinity for the ligands might determine, in PD, a decrease in the activation of protective signaling-molecules involved in the regulation of the immune response. This factor could affect different cellular processes which are impaired in PD, thereby contributing to the development of gut dysbiosis [118].

## 3. Functions and Dysfunctions of Smell

### 3.1. Olfactory System

Olfactory perception starts at the level of the olfactory epithelium in the roof of the nasal cavity. Olfactory receptor neurons (ORN) send their axons towards the olfactory bulbs. ORN carry olfactory receptors (OR; approximately 400 different OR can be expressed by humans). In the olfactory bulb ORN axons synapse with second order neurons, the mitral cells. All ORN carrying the same OR converge in the same glomerulus in the bulb. Axons from the mitral cells form the olfactory tract which directly connects to the primary olfactory cortex (including piriform and entorhinal cortices or the amygdalae). The secondary olfactory cortex includes structures such as the hippocampus, the anterior insula or the orbitofrontal cortex [122].

The olfactory system has 3 major functions [18]: (1) food intake control (e.g., localization, appetite), (2) social communication (e.g., reproductive behavior, detection of fear-related cues, recognition of kin, recognition of disease), and (3) detection of danger (e.g., toxicity, fires, prevention of food poisoning).

### 3.2. Smell Dysfunction in Neurogenerative Disease (Overview)

Impaired olfaction has been associated with a variety of age-related neurodegenerative conditions that impair cognitive and motor function, including PD [123,124], AD [125], and Huntington’s disease [126]. Smell loss may therefore be considered an important contribution to the diagnosis of neurodegenerative diseases. In PD, olfactory loss has been extensively studied and is now widely acknowledged as one of the major non-motor symptoms of the disease which precedes the occurrence of clinical motor symptoms [127]. A variety of psychophysical methods were used to evaluate orthonasal olfactory function in PD, mainly based on the identification of suprathreshold odors (UPSIT [128]) or on an comprehensive approach divided into a threshold, a discrimination, and an identification part, with the last two being suprathreshold odors (Sniffin’ Sticks [129]). Olfactory disturbances are found in around 90% of patients with PD [11] and have been considered as a supportive criterion in clinical PD diagnosis according to the International Parkinson’s Disease and Movement Disorder Society diagnostic criteria [130]. The majority of PD patients with smell loss are already functionally anosmic or severely hyposmic at the time of testing regardless of the type of olfactory test being used for diagnosis. Wenning et al. [131] presented data suggesting that olfactory function is differentially impaired in distinct Parkinsonian syndromes. They reported a preserved or mildly impaired olfactory function to be more likely for atypical parkinsonism such as multiple system atrophy, progressive supranuclear palsy, or corticobasal degeneration, whereas markedly pronounced olfactory loss appeared to suggest PD. Similar results were reported by Müller et al. [132] and Krismer et al. [133]. In dementias, the loss of smell is usually very severe. This applies to Lewy body disease (LBD), in which significant olfactory deficits were found [134,135] which does not allow differentiation from PD. Similar olfactory deficits have been shown in AD. In a meta-analysis by Mesholam et al. [125], olfactory deficits in patients with AD and PD were relatively uniform although there was a trend toward better performance in AD patients on threshold tests compared to odor identification tests. Smell loss can be observed in patients with mild cognitive impairment [136] and is associated with the progression from MCI to AD [137]. Huntington´s disease patients present with moderate hyposmia affecting olfactory detection threshold, odor discrimination and odor identification [126]. Deficits in odor identification are prevalent prior to diagnosis of HD [138]. In patients with cerebellar ataxia, olfactory impairment was found in Friedreich’s ataxia [139] and spinocerebellar ataxias [140,141,142]. Mild olfactory impairment has also been demonstrated in motor neuron disease [143,144].

### 3.3. Smell Impairments as a Biomarker for Early Onset, Progression, Cognitive Decline and Differential Diagnosis in PD

Support for the existence of a prodromal phase of PD, including a long pre-motor phase, comes from imaging, neuropathology, and various clinical or epidemiological surveys. Loss of smell is recognized as a very early non-motor symptoms of PD and has been suggested as a possible biomarker [145]. Several population-based studies already pointed out the association between unexplained smell loss and later development of PD. Our data of a large, thoroughly diagnosed patient cohort study of a Smell and Taste Clinic suggest a 10% rate of PD development among patients with diagnosed idiopathic olfactory loss [146]. The duration of the hyposmic phase prior to PD diagnosis is still a matter of debate. In many previous studies investigating the prospective risk for PD in relation to baseline [147,148,149] follow-up periods ranged from 2 to a maximum of 8 years. We could demonstrate that the olfactory dysfunction frequently precedes the PD motor symptoms by more than 10 years [146]. Accordingly, other authors indicated an incident of PD beyond 5 years of follow-up [150,151]. Additionally, studies that used retrospective patients’ self-reports reported the onset of olfactory dysfunction on average more than 10 years before PD diagnosis [76,152]. Other authors assumed that this period may last up to decades [153]. In a follow-up study of patients with idiopathic REM sleep behavior disorder who phenoconverted to PD or dementia, olfactory loss was the first marker to develop, with predicted onset >20 years before phenoconversion [154].

Furthermore, current studies indicate a correlation between olfactory function and progression of the disease as measured by motor and other non-motor symptoms. An association between disease severity and smell loss [50,155,156,157,158] and a disease duration-related progression of olfactory loss [157] might suggest the use of olfactory function as potential marker of PD progression. This was confirmed by an imaging study using Dat-SPECT, indicating that a more pronounced olfactory dysfunction was associated with greater loss of nigrostriatal dopamine neurons [159]. Additionally, non-motor symptoms such as cognitive impairment, depression, anxiety and sleep disturbances which are typically related to PD severity are associated with the degree of olfactory loss [155,159,160]. The close correlation between smell function and cognitive impairment is reflected by the results of the Parkinson’s Progression Markers Initiative study [161] which indicated that olfactory loss is one of the strongest clinical predictor of cognitive impairment in the first 2 years after PD diagnosis. Decline in cognition seems to be linked to progressive cholinergic denervation in PD as described by Bohnen et al. [162] who found a positive correlation between odor identification performance and forebrain cholinergic pathway integrity in PD patients.

### 3.4. Neuropathology of Smell Dysfunction in PD

The olfactory system could be one of the peripheral sites where PD first develops [4]. However, there is little and inconsistent information on changes at the olfactory periphery. While α-synuclein aggregates (Lewy bodies and neurites) have been described in the olfactory bulb (OB) at early neuropathological stages of the disease, α-synuclein was not detected in olfactory epithelium biopsies of PD patients [163], it was found however, in olfactory cells in PD autopsy cases [164]. Further, in-vivo examinations of the olfactory epithelium revealed histological changes comparable to other causes of smell loss [163] which suggest non-specific peripheral changes in the olfactory system in PD. On the OB level, PD seems to differ from other causes of olfactory loss. In etiologies involving peripheral olfactory loss, such as postinfectious or sinonasal smell disorders [165,166], but also in more central pathologies such as depression [167], schizophrenia [168], and temporal lobe epilepsy [169], a clear and consistent correlation between olfactory function and OB volume can be observed. These data suggest that smell loss is associated with a measurable OB volume loss in these pathologies. In PD, however, despite of the severity of olfactory impairment it remains a matter of debate whether PD patients present with decreased OB volumes compared to age-matched controls. So far, a number of recent studies have reported conflicting results: while some studies [170,171] reported an overall reduction of the OB volume in PD, the vast majority of studies [172,173,174,175] question any OB volume differences between PD and HC. This is in line with findings of an increased number of olfactory dopaminergic periglomerular cells in PD patients [176,177] which might underlie hyposmia in PD patients. However, in central regions related to both primary (piriform cortex, amygdala) and secondary integrative (orbitofrontal cortex) olfactory processing, a significant atrophy was found in PD which correlated with olfactory performance [178,179,180]. This might suggest that central olfactory areas in PD seem to represent the degree of disease progression, whereas this correlation is not seen in peripheral olfactory structures.

### 3.5. Molecular and Genetic Mechanisms Involved in Olfactory Deficit in PD

The causes of olfactory dysfunction in PD are poorly understood, but it is supposed that they are related with both peripheral and central olfactory impairments [181]. The mechanisms implicated in the smell impairments in PD may involve neuropathological alterations and/or dysfunctions caused by alteration in the neurotransmitter levels [182]. The importance of these mechanisms is addressed in the following paragraphs.

The olfactory system is one of the earliest brain regions involved in PD before involvement of the nigrostriatal pathway [4,183]. The α-synuclein deposition, predominant component of Lewy bodies [184,185], have been identified before in the olfactory bulb, anterior olfactory nucleus, and several areas of olfactory cortices of PD patients [186,187], than in the substantia nigra [4,188]. The α-synuclein pathology appears before in the olfactory nerve layer and then it spread to the central olfactory structures [189]. However, the involvement of the olfactory epithelium on olfactory loss in PD have not been well defined. In fact, no significant difference was found by immunohistochemical markers for α-synuclein between PD patients and HC [163,190]. These findings suggested that the changes in the olfactory function in PD may be due to processes associated with formation of Lewy bodies in the central olfactory areas and not in the peripheral ones [124,191]. The α-synuclein pathology has been revealed across the central olfactory system, including the anterior olfactory nucleus, cortical nucleus of the amygdala, piriform cortex, olfactory tubercle, entorhinal cortex, and orbitofrontal cortex [191,192]. In particular, the Lewy pathology in the anterior olfactory nucleus of the olfactory bulb is correlated with neuronal loss [193]. Furthermore, the olfactory nerves were grossly atrophic in all PD patients [193]. The cortical nucleus of the amygdala, which has major olfactory connections, have more α-synuclein pathology and neuronal loss than other nuclei in the amygdala [194], consequently its volume is reduced by 20% [194]. Moreover, the reduced volume in the amygdala and piriform cortex inversely correlates with olfactory deficits, suggesting that neural loss in these regions could play a role on the olfactory impairments of PD [178,179].

Several neurotransmitter systems are altered in PD and most of them have been associated with olfactory loss, including dopaminergic, cholinergic and serotoninergic systems. Dopamine has long been known to play a key role in the pathogenesis of PD. Some studies suggested that the olfactory dysfunction of PD patients could reflect damage to dopaminergic cells [195]. As a matter of fact, correlations between odor identification tests (UPSIT scores) and a decrease in dopamine transporter activity in the striatum, substantia nigra and hippocampus in PD patients have been found [50,196,197]. However, the use of dopaminergic replacement therapy has no effect on olfactory test scores [198,199]. Nevertheless, it is still not known whether changes in dopamine activity are directly associated with olfactory loss or whether there is an unknown common underlying mechanism. Acetylcholine levels are also altered in PD. It is known that acetylcholine release and activation of its receptors facilitate olfactory learning, memory, and odor discrimination [200,201,202,203]. Thus, cholinergic deficits may be responsible, at least in part, for the olfactory dysfunction in PD. It has been found that in PD the Lewy bodies and neuronal loss in the substantia nigra occur simultaneously with accumulation of the α-synuclein deposition in cholinergic neurons of the basal forebrain [4,204,205,206]. Furthermore, the nucleus basalis, a main cholinergic nucleus with projection to olfaction-related brain regions, is significantly damaged in PD [207,208,209]. In addition, Bohnen and colleagues found a positive association between odor identification performance and acetylcholinesterase activity in PD patients [162]. Serotonin is another neurotransmitter with a possible role in the pathogenesis of olfactory dysfunction in PD. It arises from the raphe nuclei, which send projections to the olfactory bulb [210,211,212]. In PD patients, Lewy pathology is found in the raphe nuclei [213], parallel to marked depletion of serotonin in the olfactory bulb and other areas of the olfactory system [214,215,216], while a relative protection of serotonin was found in other diseases without important olfactory impairments [217]. Although the evidence is not conclusive so far, these studies suggest that changes in levels of some neurotransmitters may be implicated in the olfactory loss in PD.

Polymorphisms of specific genes coding for membrane receptors or odorant binding proteins (OBPs) (carrier proteins that vehicle the molecules toward receptor sites [218]), have been reported as mechanisms that result in the functional variations of olfactory function [219,220,221,222]. Recently, the polymorphism *rs2590498* (A/G) of the *OBPIIa* gene has been shown to affect retronasal [219] and olfactory [223] perception. Subjects with the A allele were generally more sensitive than those with the G allele. Moreover, bioinformatics data suggested that the presence of the mutation in this locus decreases the expression of OBPIIa protein in the olfactory epithelium [224]. The same polymorphism affected the olfactory performance of woman with PD [224]. Specifically, the olfactory performance of women with PD carrying two sensitive alleles (AA) was higher than that of women with PD with at least one insensitive allele (G) and of all men with PD. Interestingly, the olfactory scores of the AA genotype women with PD were not different from those of HC participants. These findings indicate that the AA homozygous condition in this locus preserves the olfactory function of women with PD, but not that of men. Furthermore, these results indicate that the smell dysfunction related to PD may occur, at least in part, at a peripheral level. Therefore, *OBPIIa* locus may provide a mechanism to determine the risk factor for olfactory deficits in woman with PD at the molecular level.

### 3.6. Microbiota and Olfactory Deficit in PD

The associations between PD and the olfactory dysfunction and the composition of gut microbiota are unequivocal [225,226]. However, results on a role of the nasal microbiome in olfactory dysfunction in PD are not conclusive. A recent study has shown that there are no significant differences in the nasal microbiome composition between PD patients and HC [227]. However, the incapacity to collect samples in the olfactory cleft did not rule out the existence of differences in the microbial composition around the olfactory neuroepithelium. In addition, high spatial variability of microbial communities in the nasal cavity can exist [228]. Additionally, another study did not find consistent difference in the nasal microbiota composition between PD patients and HC, even though a high interindividual variability was observed, with sex as the strongest factor [229]. Future studies in which samples of nasal microbiome are collected in the olfactory cleft are needed to understand its role in olfactory dysfunction of PD patients. 

## 4. Conclusions

**Figure 1:**
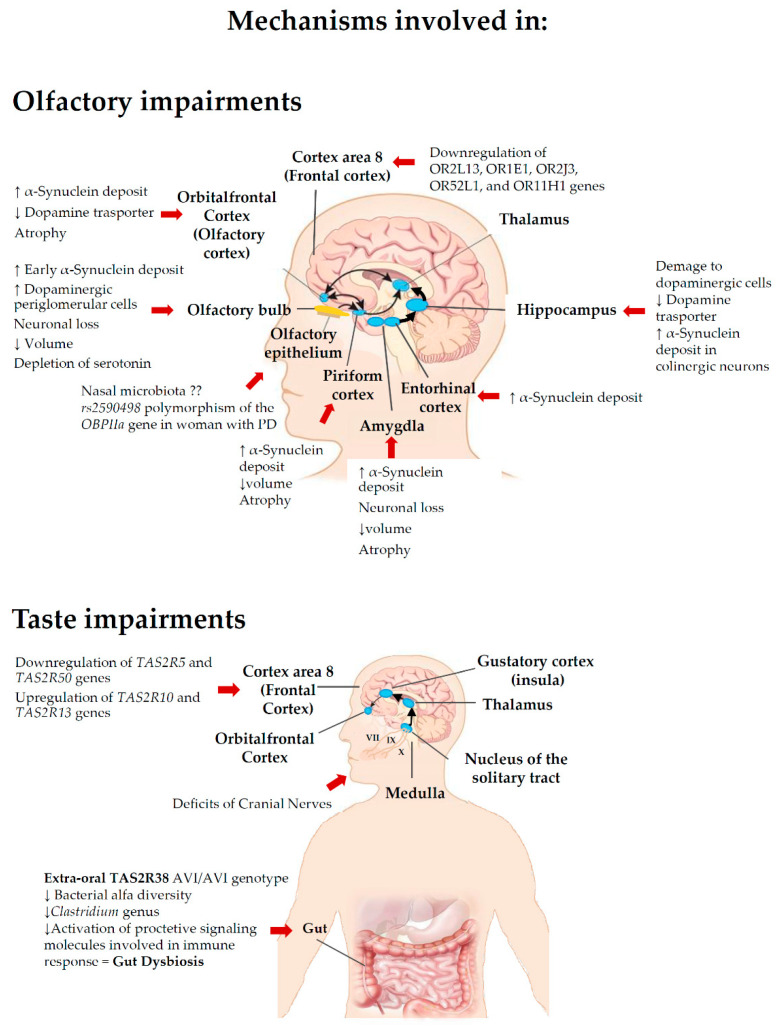
Picture showing a summary of the main mechanisms involved in olfactory and taste deficits in Parkinson’s disease.

In conclusion, this review examined the influence of smell and taste disorders in PD, describing the most relevant factors such as genetic factors, microbiome and some neuropathological mechanisms which appear to be involved in the PD-related smell and taste impairments (Figure 1). The underlying causes or their relative degree of impact are still not conclusive, thus further pre-clinical and clinical research on cellular and molecular mechanisms underlying these dysfunctions in PD are required.

Our review confirms the relevance of smell and taste in the PD patients showing that smell and taste dysfunction are non-motor manifestations of PD that may impair the quality of life. Albeit the symptom “olfactory loss” has been approved as a helpful measure in clinical PD diagnosis according to the new International Parkinson’s Disease and Movement Disorder Society diagnostic criteria [130], the use of taste as a biomarker is not yet included in the diagnosis of PD. Nevertheless, it is known that in many of the patients, taste loss accompanies smell dysfunction [146], thus testing these two sensory functions together would help clinicians in early diagnosis of PD enhancing the predictive value for diagnosis of disease.

Smell and taste impairment in PD are connected to different anatomical pathways. As mentioned above, the α-synuclein pathology involves the central olfactory system and smell impairment is often detectable years before the onset of motor symptoms of PD. Differently the pathology at the basis of taste dysfunction is less understood and the nucleus tractus solitarius is usually not involved by Lewy body pathology even in the latest stages of the disease [4]. However, such pathology does occur in the anterior insular/operculum region, which is an important relay station for axons connected to the orbitofrontal cortex [230,231]. Consequently, taste impairment in PD probably depends on the involvement of the cortex in the neurodegenerative process.

## Data Availability

No new data were created or analyzed in this study. Data sharing is not applicable to this article.
